# Cryo-EM structure of ACE2-SIT1 in complex with tiagabine

**DOI:** 10.1016/j.jbc.2024.107687

**Published:** 2024-08-17

**Authors:** Angelika Bröer, Ziwei Hu, Jędrzej Kukułowicz, Aditya Yadav, Ting Zhang, Lu Dai, Marek Bajda, Renhong Yan, Stefan Bröer

**Affiliations:** 1 Research School of Biology, Australian National University, Canberra, Australia; 2 Department of Biochemistry, Key University Laboratory of Metabolism and Health of Guangdong, School of Medicine, Institute for Biological Electron Microscopy, Southern University of Science and Technology, Shenzhen, Guangdong, China; 3 Department of Physicochemical Drug Analysis, Faculty of Pharmacy, Jagiellonian University Medical College, Cracow, Poland

**Keywords:** amino acid transport, cryo-electron microscopy, drug action, membrane transport, membrane protein

## Abstract

The pharmacology of amino acid transporters in the SLC6 family is poorly developed compared to that of the neurotransmitter transporters. To identify new inhibitors of the proline transporter SIT1 (SLC6A20), its expression in *Xenopus laevis* oocytes was optimized. Trafficking of SIT1 was augmented by co-expression of angiotensin-converting enzyme 2 (ACE2) in oocytes but there was no strict requirement for co-expression of ACE2. A pharmacophore-guided screen identified tiagabine as a potent non-competitive inhibitor of SIT1. To understand its binding mode, we determined the cryo-electron microscopy (cryo-EM) structure of ACE2-SIT1 bound with tiagabine. The inhibitor binds close to the orthosteric proline binding site, but due to its size extends into the cytosolic vestibule. This causes the transporter to adopt an inward-open conformation, in which the intracellular gate is blocked. This study provides the first structural insight into inhibition of SIT1 and generates tools for a better understanding of the ACE2-SIT1 complex. These findings may have significance for SARS-CoV-2 binding to its receptor ACE2 in human lung alveolar cells where SIT1 and ACE2 are functionally expressed.

The solute carrier 6 (SLC6) family comprises members that transport monoamines, γ-aminobutyric acid (GABA) and related compounds, and amino acids. Due to their relevance in neurotransmission, the neurotransmitter transporters in this family have a well-developed pharmacology (1, 2). The pharmacology of the amino acid transporters in this family, by contrast, is much less developed although glycine (GlyT1) and neutral amino acid transporter (B^0^AT1) are validated pharmaceutical targets (3, 4). In addition, the family comprises potential pharmaceutical targets, such as B^0^AT2 (SLC6A15), ATB^0,+^ (SLC6A14) and SIT1 (SLC6A20), which are genetically associated with depression, cancer and severity of Covid-19 infection, respectively (5, 6, 7, 8, 9, 10, 11). Thus, a better understanding of the pharmacology of this group of transporters is warranted.

A notable feature of some SLC6 amino acid transporters (B^0^AT1, B^0^AT3, and SIT1), is the formation of a heterodimeric complex with the SARS-CoV-2 receptor angiotensin-converting-enzyme 2 (ACE2) (12, 13). The complex is required for trafficking to the cell surface and functional activation of B^0^AT1, B^0^AT3 (SLC6A18), and potentially SIT1 (14, 15, 16), but not of the related transporters B^0^AT2 and ATB^0,+^ (17, 18). ACE2 is a membrane-bound peptidase involved in the breakdown of angiotensin (II), the processed hormone that regulates blood pressure. ACE2 is expressed on the surface of many cell types, but in a variety of epithelial cells it forms a complex with membrane transporters (19). The association between ACE2 and the intestinal amino acid transporter B^0^AT1 (SLC6A19) was discovered through a rare mutation resulting in the protein malabsorption syndrome known as Hartnup disorder (14). The structure of the B^0^AT1/ACE2 complex was resolved through single particle cryo-EM (20). The related transporter SIT1 is found in a wider variety of epithelial cells including the brain (21). The epithelial localization of SIT1 is highlighted by mutations associated with a rare benign aminoaciduria known as iminoglycinuria (22). The structures of the complex between SIT1 and ACE2 bound with different substrates have recently been determined by cryo-EM (23, 24, 25).

Structural studies of SIT1 show that the amino acid substrate is sandwiched by two critical residues, namely Tyr21 and Phe250 in transmembrane helices (TM) 1 and 6, respectively. The SIT1 substrate proline forms hydrogen bonds with several residues in its vicinity, namely Tyr21, Ser251, Phe250, Ser406 and Asn410. The structure also revealed the position of a chloride ion that is co-transported with proline (24). Mechanistic studies demonstrated a 2Na^+^/1Cl-proline symport mechanism (21, 26) and the position of the chloride ion is consistent with previous predictions of the ion binding sites in SIT1 (27). Despite its role in human physiology and disease, inhibitors of SIT1 have not been identified. The transporter does accept close structural analogues of proline such as nipecotic acid and pipecolic acid (21), which can serve as low-affinity inhibitors of the transporter. Nipecotic acid is also an inhibitor of GABA uptake (28) pointing to a close relationship of the active site of SIT1 to GABA transporters. A cryo-EM structure of the human GABA transporter hGAT1 in complex with tiagabine revealed an unexpected binding mode in an inward-open state of the transporter (29), while modelling of previously generated data suggested binding to an outward-open state (30).

In this study, we aimed to identify new inhibitors of SIT1 and investigated whether ACE2 enhances expression of SIT1 in *Xenopus laevis* oocytes to optimize its use for pharmacological studies. We identified nipecotic acid-based compounds as inhibitors of the ACE2/SIT1 and determined the structure of SIT1 in the presence of tiagabine by single particle cryo-EM and detected ACE2 and SIT1 in human alveolar cells.

## Results

### ACE2 functionally interacts with SIT1 in *X. laevis* oocytes

**Figure 1:**
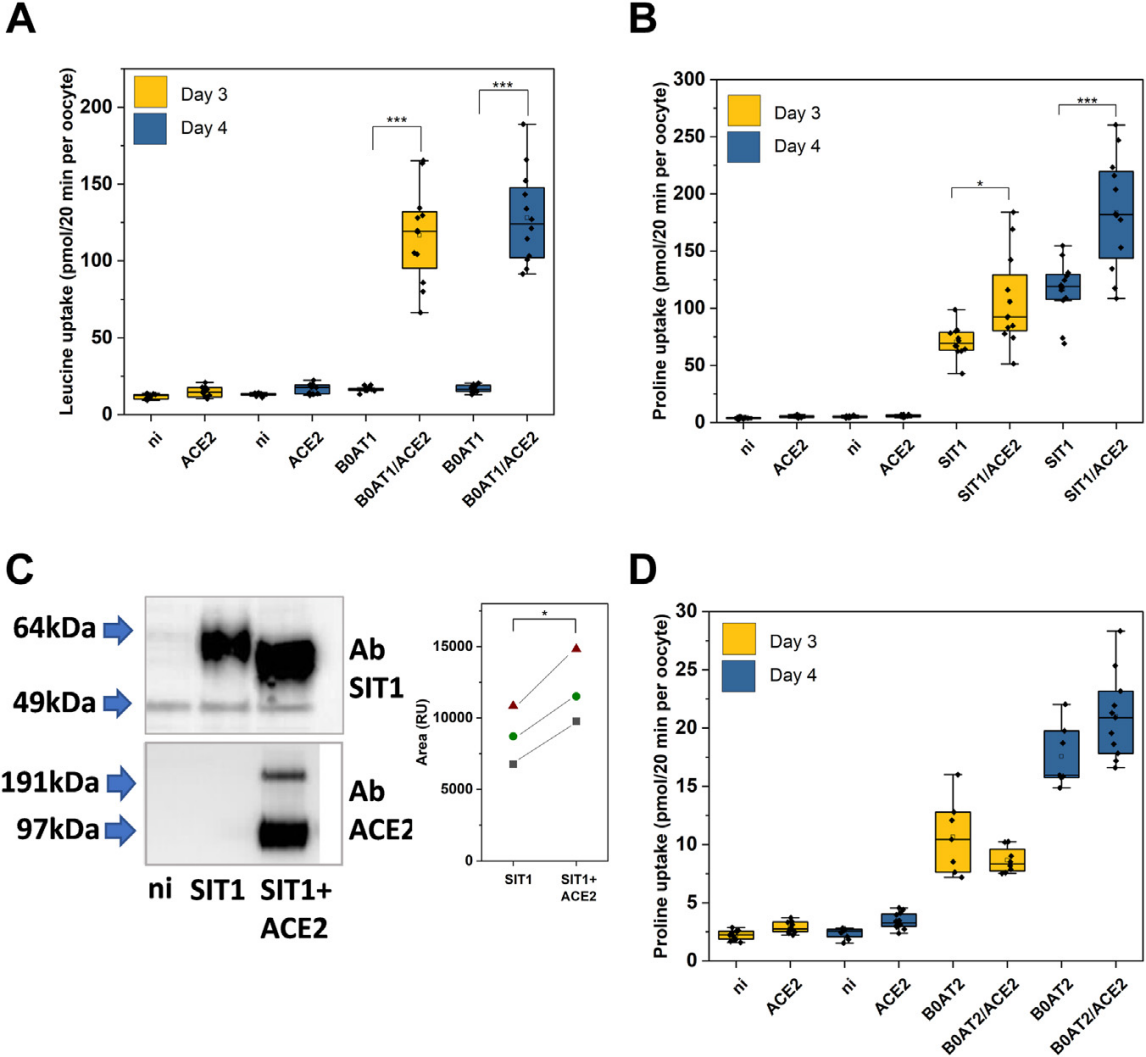
**Modulation of SIT1 activity by ACE2.***Xenopus laevis* oocytes were injected with cRNA for SIT1 (5 ng) B^0^AT1 (5 ng), B^0^AT2 (5 ng) and ACE2 (20 ng) alone or in combination or remained uninjected (n.i.) and were incubated for 3–6 days. *A*, uptake of [^14^C] leucine in oocytes expressing B^0^AT1 and/or ACE2 on day 3 and day 4 (n = 11–12 biological replicates). *B*, uptake of [^14^C] proline in oocytes expressing SIT1 and/or ACE2 on day 3 and day 4 (n = 12 biological replicates). *C*, detection of SIT1 and ACE2 in oocytes after 6 days of expression of SIT1 alone or in combination with ACE2 (n = 3 biological replicates, quantification in side panel, effect of ACE2 *p* = 0.01 paired *t* test). *D*, uptake of [^14^C]proline in oocytes expressing B^0^AT2 and/or ACE2 on day 3 and day 4 (n = 7–12 biological replicates). Distribution of data (median, 25 and 75 percentile and SD) shown, one-way ANOVA *p*-values shown as ∗<0.05 and ∗∗∗<0.001 (Scheffé *post hoc* test).

To improve the pharmacology of SIT1, we have used *X. laevis* oocytes as an expression system. Previously, we expressed SIT1 alone (21), but there is increasing evidence that ACE2 may augment expression of SIT1 (12). As a result, we investigated the interaction between ACE2 and SIT1 and compared it to other transporters in this family. Both proteins can be expressed individually and the trafficking of the transporter to the surface can be analysed by transport activity. In oocytes, surface expression of the closely related transporter human B^0^AT1 (SLC6A19) was fully dependent on the co-expression of ACE2 (Fig. 1*A*) agreeing with previous reports (14). The activity of SIT1 was augmented by coexpression of ACE2, but the transporter was also active in the absence of ACE2 (Fig. 1*B*). Western blotting confirmed the surface expression of SIT1 and its increase upon co-expression of ACE2 in *X. laevis* oocytes (*p* = 0.01). Notably, western blotting indicated a shift of the SIT1 molecular weight in the presence of ACE2 (Fig. 1*C*). These data suggest an interaction between SIT1 and ACE2 in oocytes. As a negative control we expressed the related transporter human B^0^AT2 (SLC6A15), the activity of which was unchanged when co-expressed with ACE2 (Fig. 1*D*). As a result, we performed further experiments in the presence of ACE2.

#### Tiagabine (TGB) is an inhibitor of SIT1

**Figure 2:**
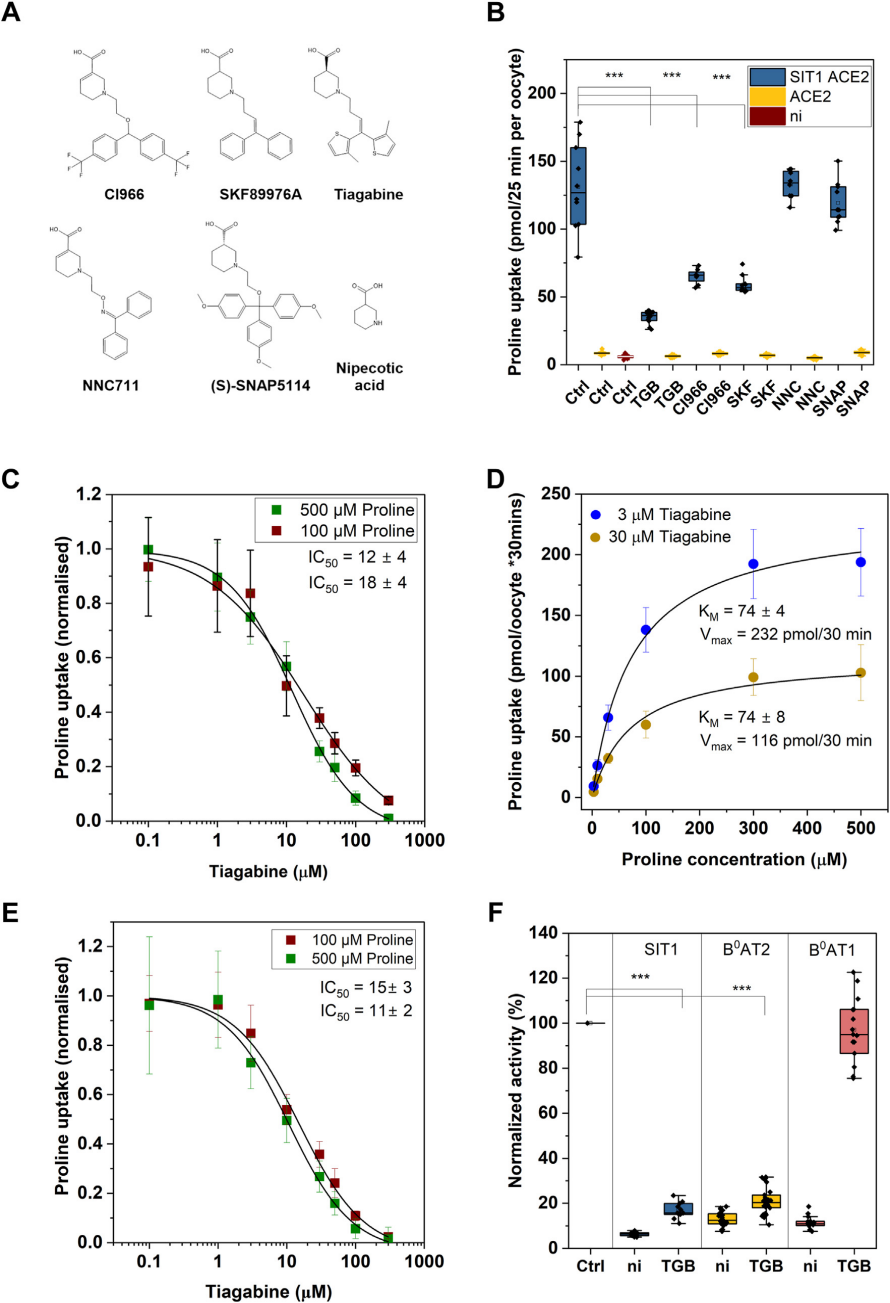
**Inhibitors of SIT1.***A*, structures of GABA transport inhibitors. *B*, oocytes were injected with 15 ng SIT1 cRNA and/or 15 ng ACE2 cRNA or remained uninjected (ni). After incubation for 4 days proline uptake activity (100 μM [^14^C]proline) was determined in the presence and absence of GABA transport inhibitors (100 μM) (n = 8–12 biological replicates). *C*, dose-response curve for tiagabine at 100 μM and 500 μM [^14^C]proline in oocytes expressing SIT1-ACE2 (n = 9–15 biological replicates, error bars represent S.D.). *D*, kinetic parameters of proline transport in oocytes expressing SIT1-ACE2 (n = 8–14 biological replicates, error bars represent S.D.). *E*, dose-response curve for tiagabine at 100 μM and 500 μM [^14^C]proline in oocytes expressing SIT1 alone (n = 9–15 biological replicates, error bars represent S.D.). *F*, oocytes were injected with cRNA encoding SIT1 (15 ng), B^0^AT1 (10 ng) B^0^AT2 (12.5 or 25 ng) and ACE2 (1:1 ratio to SIT1 and B^0^AT1) or remained uninjected (ni). Activity was measured by uptake of 100μM labelled substrate (leucine for B^0^AT2, proline for SIT1) in the absence and presence of tiagabine (100 μM) (n = 11–14 biological replicates). Distribution of data (median, 25 and 75 percentile and SD) shown, one-way ANOVA *p*-values shown as ∗∗∗<0.001 (Scheffé *post hoc* test).

To identify potential inhibitors of SIT1 we screened GABA transporter inhibitors tiagabine (GAT1), SKF89976A (GAT1), CI966 (GAT1), NNC711 (GAT1), and (S)-SNAP 5114 (GAT2,3) because nipecotic acid is a fragment of these inhibitors and a substrate of GAT and SIT1 (Fig. 2*A*).

Tiagabine stood out as a potent inhibitor of SIT1 (77% inhibition at 100 μM). CI966 (53% inhibition) and SKF89976A (58% inhibition) were less potent, while NNC711 and (S)-SNAP 5114 were ineffective (Fig. 2*B*). A more detailed analysis revealed an IC_50_ of 18 ± 4 μM for tiagabine at 100 μM proline and of 12 ± 4 μM at 500 μM proline (difference *p* = 0.2; Fig. 2*C*). Modelling of the IC_50_ using equations for competitive, non-competitive and uncompetitive inhibition suggested that tiagabine was not a competitive inhibitor (Table 1) but the variance of the data did not allow discrimination between non-competitive and uncompetitive modes of inhibition.

**Table 1:** Experimental and predicted IC_50_-values of inhibition of SIT1 by tiagabine

Inhibition type	100 μM proline	500 μM proline
Experimental	18 ± 4	12 ± 4
Uncompetitive model	28	15
Non-competitive model	12	12
Competitive model	21	58

Inhibition of proline transport by tiagabine was modelled using equations for competitive, non-competitive and uncompetitive inhibition with the following constants: K_M_ = 130 μM, V_max_ = 100, Substrate concentration (100 or 500 μM), K_i_ = 12 μM.

Analysis of the K_M_ and V_max_ at two different concentrations of tiagabine showed a change of the V_max_ without affecting the K_M_ (Fig. 2*D*). This behavior is consistent with a non-competitive mode of inhibition. Tiagabine also inhibited SIT1 when expressed in the absence of ACE2 (Fig. 2*E*) with IC_50_ values of 15 ± 3 μM and 11 ± 2 μM at 100 μM and 500 μM proline, respectively. This result is also more consistent with a non-competitive mode of inhibition. The branched-chain amino acid transporter B^0^AT2 (SLC6A15) was similarly sensitive to inhibition by Tiagabine, but not the neutral amino acid transporter B^0^AT1 (Fig. 2*F*).

#### Structural characterization of the ACE2-SIT1 complex bound to tiagabine

**Figure 3:**
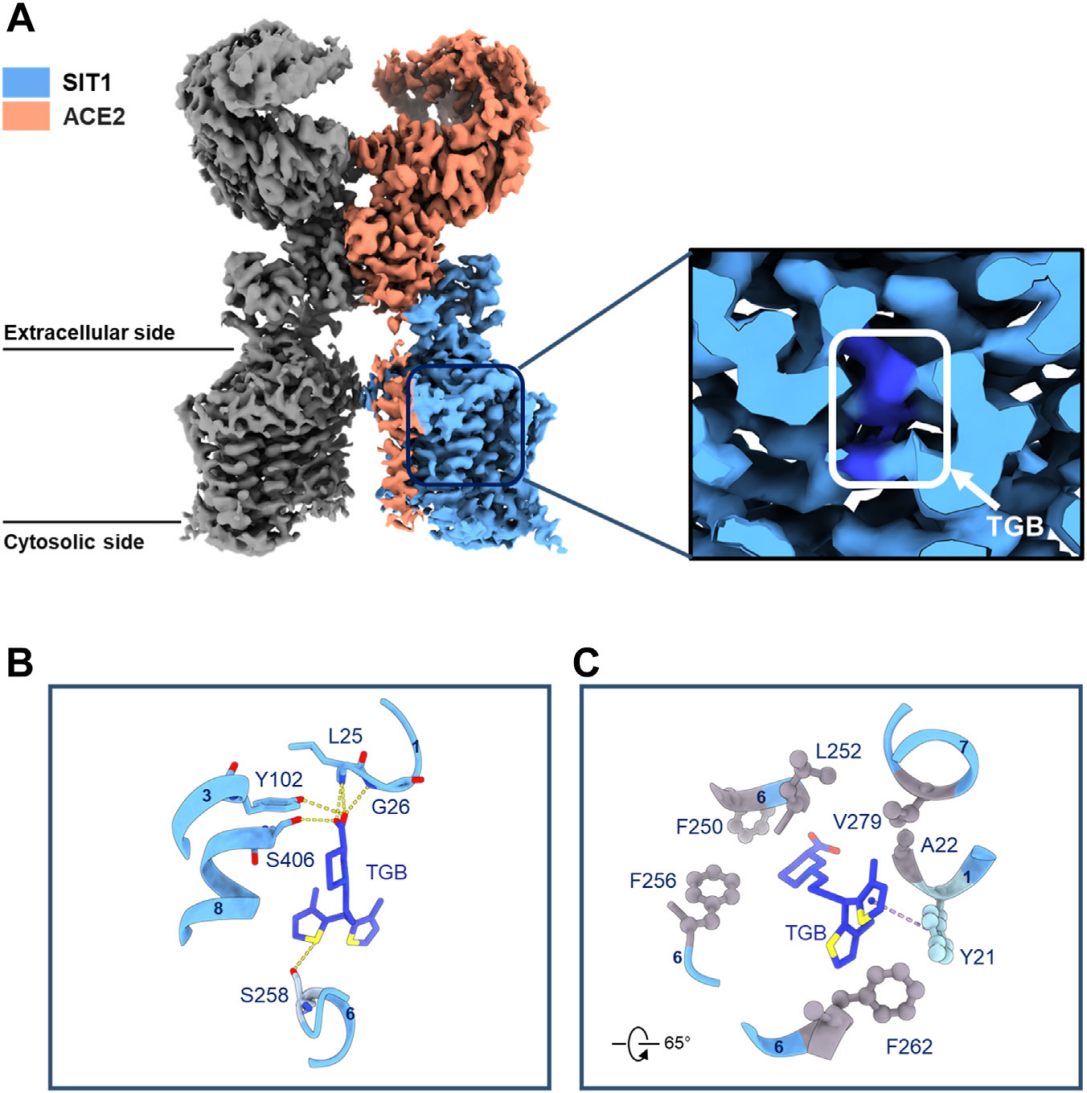
**Overall structure of the ACE2-SIT1 bound with tiagabine (TGB).***A*, Cryo-EM map of the full length of ACE2-SIT1 complex with tiagabine (TGB). ACE2 and SIT1 bound with tiagabine (*royal blue*) are represented in *light coral* and *light sky blue*, respectively. In the insert of the Cryo-EM map of the human ACE2-STI1 complex, the density corresponding to tiagabine is color-coded in *royal blue*. *B*, the hydrogen bond network at the tiagabine binding pocket of the SIT1 structure. Hydrogen bonds clusters are indicated by *yellow dashed lines*. *C*, hydrophobic interactions formed among Phe250, Leu252, Phe256, Phe262, Val279, Ala22 and Tyr21.

To further study the non-competitive inhibition mode of tiagabine, we aimed to determine the cryo-EM structure of the ACE2-SIT1 complex incubated with tiagabine. After multiple rounds of refinement, we successfully resolved the structure at a resolution of 3.34 Å (Fig. S1). Notably, we observed a clear electron density for tiagabine overlapping with the orthosteric substrate binding site (Fig. 3*A*). Detailed information regarding protein overexpression, cryo-EM sample preparation, data collection and processing, and model construction can be found in the “Experimental procedures” section, Figs. S1–S3, and Table S1.

**Figure 4:**
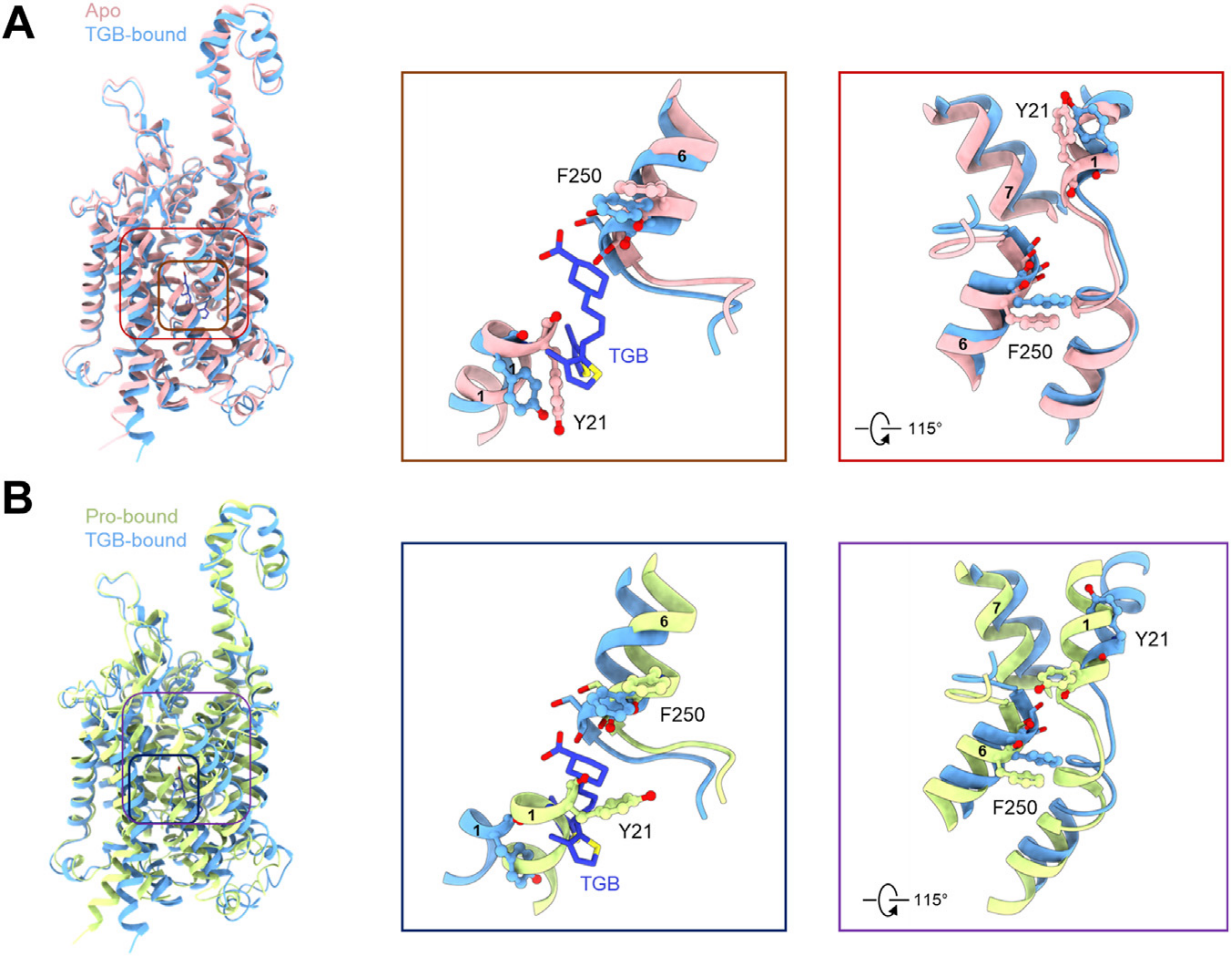
**The conformational movement of SIT1 with different states.***A*, a structural comparison is presented between the apo structure of SIT1 (PDB: 7Y75) and SIT1 bound with tiagabine. The apo structure is depicted in *pink*, while SIT1 with tiagabine is shown in *light sky blue*. The alternation in the positions of TM1 and TM6 are illustrated in the middle and right panels. *B*, structural comparison is provided for SIT1 bound with proline and tiagabine, respectively. The superimposed structures of SIT1 bound with proline are represented in *yellow green*, SIT1 with tiagabine in *light sky blue*, and tiagabine itself colored in *royal blue*.

Tiagabine forms a highly stable complex with SIT1, facilitated by a well-defined network of hydrophobic and hydrogen bond interactions. This network primarily centers around two key residues, Tyr21 and Phe250, with most interactions occurring in the proximity of transmembrane domains TM1 and TM6. Tyr21 and Phe250 form the intracellular and extracellular border of the substrate binding site, respectively. Specifically, tiagabine establishes crucial hydrogen bonds with the backbone amino groups of Leu25, Gly26, and the side chain of Tyr 102 and Ser406 (Fig. 3, *B* and *C*). Notably, we also found that the sulphur atom in the 3-methyl-2-thienyl groups can form non-covalent interactions with Ser258 in TM6. The cavity housing tiagabine is rich in hydrophobic amino acids, including Val279, Ala22, Phe250, Leu98, Phe256, and Leu252, which promote its stable occupancy within the cavity (Fig. 3*C*). Furthermore, Tyr21 engages in aromatic interactions with tiagabine, mainly through T-shaped π-π interaction (Fig. 3*C*). These residues play a pivotal role in shaping the tiagabine binding pocket. When compared to the SIT1 apo structure in an inward open conformation and the SIT1 structure bound with proline in an occluded conformation (23, 24), the tiagabine-bound structure adopts a more inward-open state, with the intracellular gating residue Tyr21 opening significantly, and the distances among TM1, TM6, and TM7 increasing in SIT1 when it binds to tiagabine (Fig. 4*A*). As a result, the intracellular gate is pried open by tiagabine. The inhibitor acts like a wedge preventing the return of the apo-transporter to the outward-open conformation. This is particularly obvious when comparing the occluded Pro-bound structure with the tiagabine bound structure (Fig. 4*B*).

**Figure 5:**
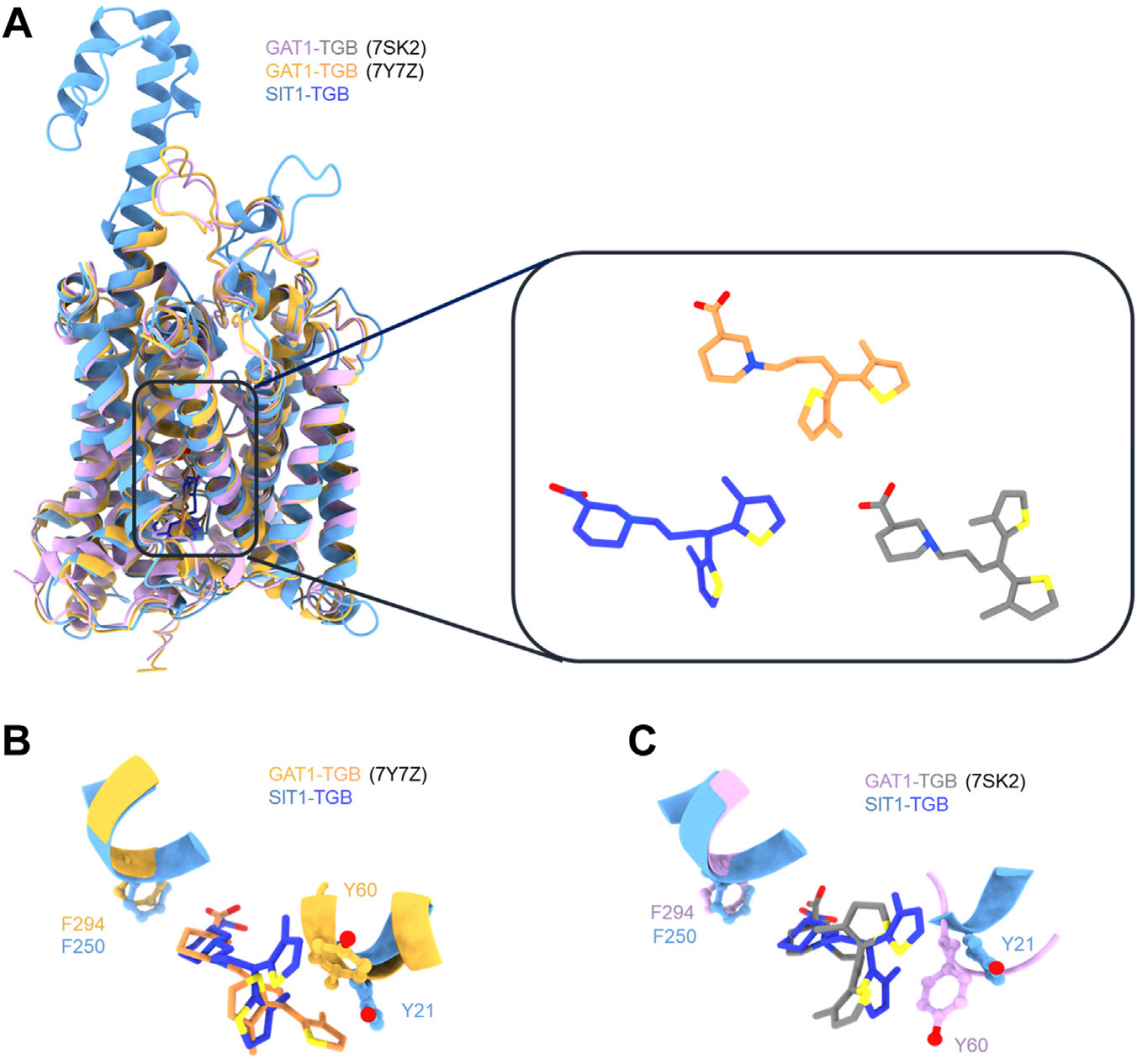
**Structural comparisonofSIT1 and GAT1 bound with tiagabine.***A*, the superimposed structures of SIT1 with tiagabine (*light sky blue*), GAT1 bound with tiagabine (PDB: 7Y7Z, *bright yellow*), and GAT1 with tiagabine (PDB: 7SK2, *light purple*). *B*, structural comparison between SIT1 and GAT1 (PDB: 7Y7Z), with tiagabine colored in *royal blue* and *orange*, respectively. *C*, structural comparison between SIT1 and GAT1 (PDB: 7SK2), with tiagabine presented in *royal blue* and *purple*, respectively. The gating residues Phe250 and Tyr21 in SIT1, corresponding to Phe294 and Tyr60 in GAT1, are depicted in ball-stick representation.

Given that tiagabine is a known GABA transport inhibitor, we conducted a comparative analysis between SIT1 and GAT1 when bound to tiagabine (PDB: 7Y7Z (31) and 7SK2 (29)). Notably, although tiagabine is coordinated in the center of two gating residues (Tyr21 and Phe250 in SIT1 and Phe294 and Tyr60 in GAT1), the position of the gating residue Phe250 exhibits slight differences, while Tyr21 shows a notable shift, indicating a distinct mechanism of inhibition (Fig. 5).

#### ACE2 and SIT1 are expressed in human alveolar cells

**Figure 6:**
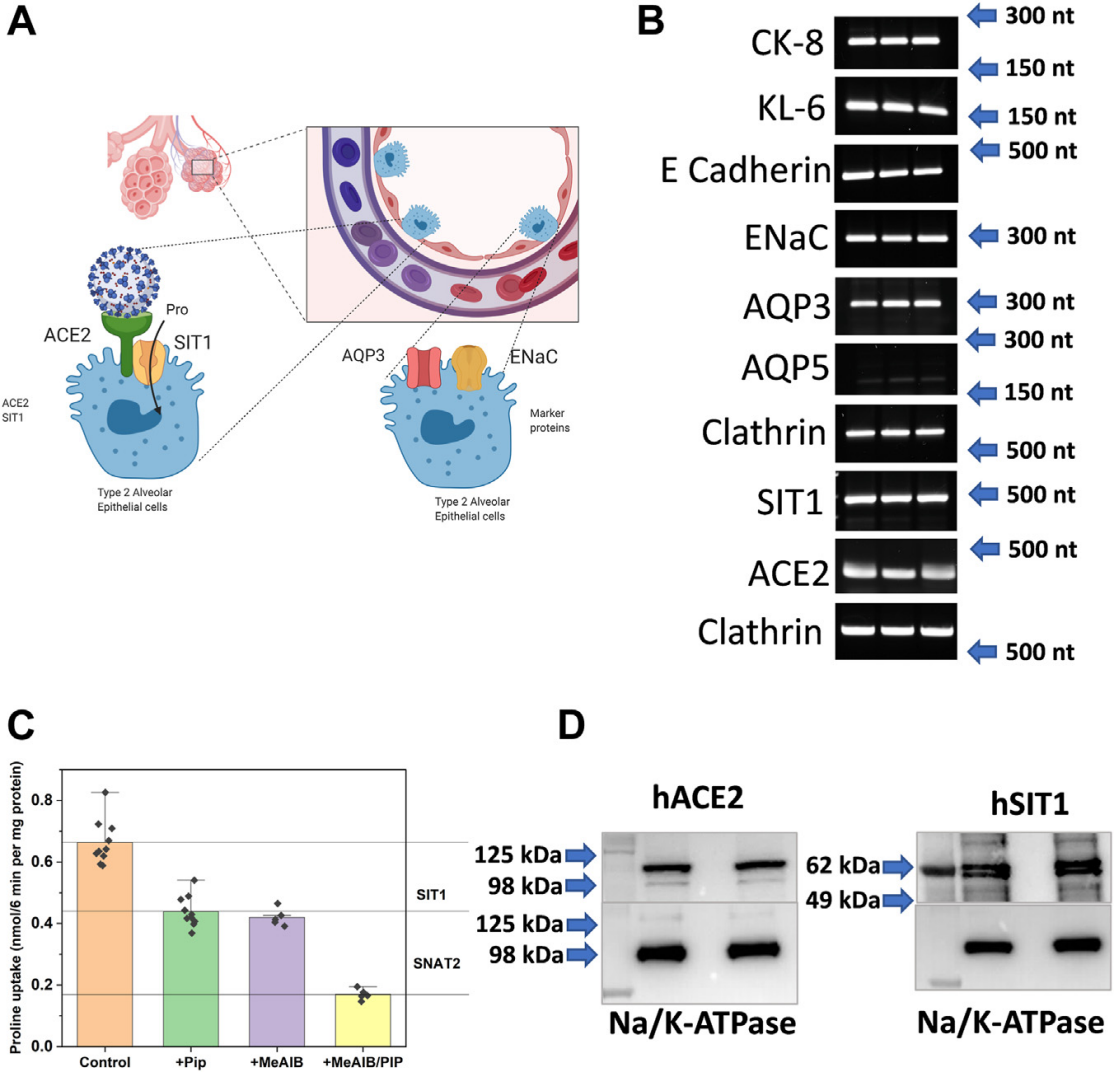
**Expression of SIT1 and ACE2 in human alveolar cells.***A*, schematic histology of lung alveoli. *B*, detection of RNA expression of markers of type 1 and type 2 alveolar cells and of SIT1 and ACE2 in primary human alveolar cells by RT-PCR (n = 3 biological replicates). *C*, transport of 100 μM [^14^C]proline was measured in the presence and absence of 10 mM pipecolate (Pip), 10 mM MeAIB and their combination to delineate different transport activities (n = 8–10 biological replicates). *D*, SIT1 and ACE2 were detected by western blotting in human alveolar cells. Membrane proteins were enriched by surface biotinylation and binding to streptavidin beads (samples are separated by an empty lane). Na,K-ATPase was used as a loading control (n = 2).

Because the SIT1 gene is located in a genomic region associated with the severity of COVID-19 infection and we previously detected SIT1 expression in mouse lungs (21), we wanted to investigate the presence of both proteins in human alveolar cells. Using the human protein atlas (www.proteinatlas.org), we confirmed the expression of SIT1 and ACE2 RNA in alveolar cells (normalized single-cell RNA count >1.0 nTPN). Lung alveoli contain two different types of epithelial cells, namely thin type I cells optimized for gas exchange and large cuboidal type II cells with an apical brush border (32) (Fig. 6*A*). Type II cells optimize the alveolar environment through surfactant secretion and fluid retention. These cell types can be discriminated by marker proteins, with aquaporin 5 (AQP5) being expressed in type I cells, while cytokeratin 8 (CK-8), aquaporin 3 (AQP3), mucin KL-6, E-cadherin and the epithelial sodium channel (ENaC) are expressed in type II cells. In primary cultures of human alveolar cells, we could detect mainly type II cells (Fig. 6*B*). These cells also expressed significant transcripts for SIT1 and ACE2 (Fig. 6*B*). To see whether we could identify functional SIT1 in type II cells, we analysed the transport activity of human alveolar cells. Proline uptake was mediated by SNAT1/2 (SLC38A1/2) and SIT1 as indicated by partial inhibition of transport in the presence of N-methylaminoisobutyric acid (MeAIB), inhibiting SNAT1/2 (33) and pipecolate, inhibiting SIT1 (21) (Fig. 6*C*). The inhibitory effect of pipecolate was additive to that of MeAIB, suggesting expression of proline transporters SNAT1/2 and SIT1 in these cells. Surface biotinylation followed by enrichment on streptavidin beads confirmed the expression of SIT1 and of ACE2 on the surface of type 2 cells (Fig. 6*D*). SIT1 displayed a double-band, resembling the shift of molecular weight observed after coexpression of ACE2 with SIT1 in *X. laevis oocytes* albeit only in a fraction of the protein population. The double band is unlikely to result from masking of a glycosylation site, which in the structure are found at Asn 131, 332 and 357, the former two of which are predicted by sequence analysis (Uniprot database).

## Discussion

This study makes two significant advances. First, we identified tiagabine as an inhibitor of amino acid transporters in the SLC6 family, which has implications for its pharmacological actions. Secondly, we present the first inhibitor-bound high-resolution structure of the ACE2-SIT1 complex, which may have implications for its role in epithelial cells in the brain, lung, intestine, and kidney.

Nipecotic acid is the core of the tiagabine molecule and is a substrate of SIT1 and GAT1. Consistently, the nipecotic acid fragment of tiagabine is in the binding site of SIT1, extending into the intracellular vestibule. Tiagabine discriminates >10,000-fold against other GABA transporters, while the discrimination towards SIT1, depending on the experimental system, is only 10 to 200-fold (31, 34). Thus, pharmacological doses of tiagabine could inhibit SIT1, which is also expressed in brain tissue (21). Notably IC_50_-values measured in the oocyte expression system are often higher than those determined in cultured mammalian cells (35), thus inhibition of SIT1 in brain cells might be even more prominent. In GAT1, mutation of Tyr60 has a major impact on the binding of tiagabine (29). This residue is equivalent to Tyr21, which coordinates tiagabine in SIT1. Motiwala *et al.* (29) suggested a two-step mechanism for inhibition of GAT1 by tiagabine. This was based on a competitive mode of inhibition when tiagabine was used without preincubation and a non-competitive mode of inhibition after preincubation. The authors proposed that tiagabine was acting as a substrate analogue being translocated through the transport pore. Given the size of tiagabine, it appears unlikely that the transporter could form an occluded transition state around the inhibitor. It appears more likely that tiagabine can enter this site in GAT1 from the cytosol, which also would explain the change of inhibition mode after preincubation. Most likely tiagabine could bind to the outward-open conformation of GAT1 in a competitive manner and to the inside open conformation in a non-competitive manner in intact cells. The intracellular site of GAT1 is readily accessible in the solubilized complex and appears to be the preferred binding site in this state. However, molecular dynamics and mutational studies also support binding from the outside (30), which is further supported by rapid inhibition and lack of accumulation of tiagabine in synaptosomes (36). In our experiments tiagabine inhibited SIT1 with a non-competitive mode, excluding a competitive mode of inhibition. Binding of tiagabine to the cytosolic face of the transporter could kinetically appear as a non-competitive mechanism because competition with the substrate would occur on the cis-site of the transporter.

Our results suggest that pharmacological doses of GAT1 inhibitor tiagabine could affect additional transporters in the brain, notably SIT1 and B^0^AT2. SIT1 has been suggested to regulate proline and glycine levels in the brain (37). Mice that overexpress SLC6A20 due to a mutation in PTEN showed decreased glycine levels and NMDA-receptor function, while SLC6A20 ko mice showed increased glycine levels and NMDA-receptor activity. B^0^AT2 ko mice show lower levels of anxiety and depressive-like behavior following chronic social stress but behave normally under basal conditions (38). The interactions we have uncovered between SIT1 and GAT1 binding properties offer valuable insights that could lead to the design of more targeted inhibitors.

The pathogenic coronavirus SARS-CoV-2 and its variants continue to pose a public health threat by causing Coronavirus Disease 2019 (COVID-19). Genome-wide association approaches have been used to identify host genes that could be involved in the severity of the infection. These studies identified chromosome 3p21.31 to be associated with severe COVID-19 cases. The region comprises several immune-related genes, such as cytokines and lymphocyte surface receptors, and SIT1 as potential candidates. Using gene editing experiments in several cell lines it was shown that expression of SIT1 may increase upon SARS-CoV-2 infection (39). A mutual interaction between ACE2 and SIT1 may thus affect infection capacity. Notably, ACE2 is restricted in its mobility when bound to SIT1. In mouse kidney, surface expression of SIT1 is abrogated in the absence of collectrin, which replaces the function of ACE2 in this organ (40). It appears likely that SIT1 surface expression in the lung is similarly reliant on ACE2 coexpression. In *X. laevis* oocytes ACE2 is not essential for active expression of SIT1 but augments it. The most likely reason for this difference is the incubation at lower temperature in oocytes, which ameliorates trafficking and folding problems occurring at human or rodent body temperature (41).

In summary, we show that tiagabine is an effective inhibitor of SIT1 and provide a structural understanding of its inhibition of this transporter. This could be relevant for the action of tiagabine as an anticonvulsant. The role of this complex for the entry of SARS-CoV2 remains to be elucidated.

## Experimental procedures

### Oocyte expression

Holding of *X. laevis* frogs (purchased from Nasco) and the surgical procedure to remove parts of the ovary were approved by the Animal experimentation ethics committee of the Australian National University (Protocol A2020/28). All procedures were carried out in accordance with the recommendations of the Australian code for the care and use of animals for scientific purposes. *X. laevis* oocytes were isolated and maintained as described previously (42). Selected oocytes were injected with cRNA encoding ACE2, B^0^AT1, B^0^AT2 and SIT1 and incubated for up to 6 days. Subsequently, uptake experiments were performed using ND96 buffer (96mM NaCl, 2mM KCl, 1.8mM CaCl_2_, 1mM MgCl_2_, 5mM HEPES; titrated with NaOH to pH7.4). For uptake experiments ND96 was supplemented with 100 μM [^3^H]proline or 100 μM [^14^C]leucine and incubated with the oocytes for the indicated time. To terminate uptake, oocytes were washed three times with 4 ml of ice cold ND96, transferred to scintillation vials, lysed with 200 μl 10% SDS, and counted for determination of accumulated radioactive.

### Protein expression and purification

The cDNAs for full-length human SIT1 (accession number: NM_020208.3) and ACE2 (accession number: NM_001371415) were subcloned into pCAG vectors separately. An N-terminal FLAG tag was fused to SIT1, while 10xHis was fused at the C-terminus of ACE2 using standard PCR techniques. The recombinant proteins were overexpressed in HEK 293F cells (Invitrogen) using SMM 293T-II medium (Sino Biological Inc) at 37 °C in a Multitron-Pro shaker (Infors) at 130 rpm under 5% CO2. When the cell density reached approximately 2.0 × 10^6^ cells/ml, the plasmids were transiently transfected into the cells. All plasmids used for transfection were prepared using the GoldHi EndoFree Plasmid Maxi Kit (CWBIO). To co-express the ACE2-SIT1 complex, approximately 0.75 mg of SIT1 plasmid and 0.75 mg of ACE2 plasmid were pre-mixed with 3 mg of polyethylenimine in 50 ml of fresh SMM 293T-II medium for 15 min before being added to 1 L cell culture. Each batch of protein preparation used 2 L HEK 293F cells. The transfected cells were cultured for 48 to 60 h before harvesting.

For purification of the ACE2-SIT1 complex, the cells were collected in a buffer containing 25 mM HEPES, pH 7.5, 150 mM NaCl, and three protease inhibitors, aprotinin (1.3 μg/ml, AMRESCO), pepstatin (0.7 μg/ml, AMRESCO), and leupeptin (5 μg/ml, AMRESCO). The membrane fraction was solubilized at 4 °C for 2 h with 1% (w/v) N-Dodecyl-β-D-maltoside (DDM) and the cell debris was removed by centrifugation at 18,700*g* for 45 min. The supernatant was loaded onto anti-FLAG M2 affinity resin (Genscript). After rinsing with the washing buffer containing 25 mM HEPES, pH 7.5, 150 mM NaCl, and 0.02% GDN (w/v), the protein was eluted from the resin with elution buffer one plus 0.2 mg/ml FLAG peptide. The eluent was further purified by Ni-NTA affinity resin (Qiagen). Wash buffer and elution buffer for the Ni-NTA resin was identical to the washing buffer mentioned above plus 10 mM and 300 mM imidazole, respectively. Subsequently, the protein complex was subjected to size-exclusion chromatography (Superose 6 Increase 10/300 Gl, GE Healthcare) in buffer containing 25 mM HEPES, pH 7.5, 150 mM NaCl and 0.02% glycodiosgenin. The peak fractions were collected and concentrated for EM analysis.

### Cryo-EM sample preparation and data acquisition

The purified ACE2-SIT1 complex was concentrated to approximately 8 mg/ml and incubated with 200 μM tiagabine for 30 min before application to the grids. Small aliquots (3.3 μl) of the protein complex were meticulously deposited onto glow-discharged holey carbon grids (Quantifoil Au R1.2/1.3). These grids were then subjected to blotting for either 3 s or 3.5 s and rapidly frozen in liquid ethane, maintaining cryogenic temperatures with liquid nitrogen, utilizing a Vitrobot (Mark IV, Thermo Fisher Scientific). Subsequently, the cryo-EM samples were transferred to a Titan Krios electron microscope operating at 300 kV. This advanced microscope was equipped with a Gatan K3 detector and a GIF Quantum energy filter. Movie stacks were autonomously acquired using AutoEMation, with an energy filter slit width set at 20 eV. Defocus levels ranged from −1.2 μm to −2.2 μm in super-resolution mode, all under a nominal magnification of 81,000×. Each stack was exposed for 2.56 s, with an exposure time of 0.08 s per frame, accumulating a total of 32 frames per stack. The total dose rate applied was approximately 50 electrons per Angstrom squared (e−/Å2) for each stack. Subsequently, the stacks underwent motion correction using MotionCor2, were binned 2-fold, resulting in a pixel size of 1.095 Å/pixel. Dose weighting was also applied, and the defocus values were accurately estimated using Gctf (43).

### Data processing

Particles were automatically extracted from micrographs that were manually selected using CryoSPARC (44, 45, 46, 47, 48). Following a round of 2D classification, a subset of high-quality particles was chosen for further processing, advancing into 3D reconstruction *via* Ab-initio Reconstruction to achieve an initial coarse resolution of the complex. For refinement to attain high resolution, the Heterogeneous Refinement method was employed. During this process, the program continually displayed the resolution, as assessed through Fourier Shell Correlation, and provided other diagnostic information for each iteration. To further enhance the map quality, particularly in the transmembrane (TM) region, non-uniform refinement and local refinement strategies were implemented. These involved applying appropriate masks to specifically target and improve the resolution in the TM region. The resolution was estimated with the gold-standard Fourier shell correlation 0.143 criterion (49) with high-resolution noise substitution (50). Refer to Fig. S1 and Table S1 for details of data collection and processing.

### Model building and structure refinement

The atomic models of the ACE2-SIT1 complex bound with tiagabine were generated based on the corresponding Cryo-EM maps. We used the previous structure of the ACE2-SIT1-BA.2 RBD complex (PDB ID: 7Y75) as a template for construction. These models underwent further manual refinement using Coot (51), with meticulous attention given to the chemical properties of each residue during the building process.

For precise refinement, we conducted structural real-space refinement employing Phenix (52). In this refinement process, we incorporated secondary structure and geometry restraints to prevent potential overfitting of the structure. To assess the model's performance and minimize the risk of overfitting, we initially refined it against one of the two independent half maps, following the gold-standard 3D refinement approach. Subsequently, we rigorously validated the refined model against the other half map.

Comprehensive statistics related to data collection, 3D reconstruction, and model refinement can be found in Table S1. Statistical tests and distribution of data points for transport experiments were calculated using MedCalc Statistical Software version 22.016 (MedCalc Software Ltd, Ostend, Belgium; https://www.medcalc.org; 2023). Where tested a one-way ANOVA with Scheffé’s *post hoc* test was used. To identify differences between IC_50_ –values a paired *t* test of the individual datapoints was used.

### Determination of IC_50_ and modelling of inhibition

IC_50_-values were calculated by non-linear regression (Origin 2022) using the equation:

$$y=\min+\left(\frac{\max-\min}{1+{\left(\frac{x}{IC50}\right)}^{n}}\right)$$

Inhibition of proline transport by tiagabine was modeled using equations for competitive, non-competitive, and uncompetitive inhibition (53):

$$y=\frac{V\;\left[S\right]}{K_{M}\;\left(1+\frac{\left[I\right]}{K_{i}}\right)+\left[S\right]}\text{competitive}$$

$$y=\frac{V\;\left[S\right]}{K_{M}\;\left(1+\frac{\left[I\right]}{K_{i}}\right)+\left[S\right]\left(1+\frac{\left[I\right]}{K_{ii}}\right)}\;\text{non}-\text{competitive}$$

$$y=\frac{V\;\left[S\right]}{K_{M}+\left[S\right]\left(1+\frac{\left[I\right]}{K_{ii}}\right)}\text{uncompetitive}$$

with the following constants: K_M_ = 130 μM, V_max_ = 100, Substrate concentration (100 or 500 μM), K_i_ = K_ii_ = 12 μM, inhibitor concentrations 0.1 to 500 μM. This was followed by curve fitting as above.

### Human alveolar cells and RT-PCR

Human alveolar cells were obtained from Creative Bioarray (CSC-C9223J) and grown in gelatin-coated culture flasks. Cells were maintained in SuperCult complete human epithelial cell medium (CM-1098X) or SAGM small airway epithelial growth medium (Lonza CC-3118) at 37 °C and 5CO_2_. Cells were seeded and expanded for 3 to 7 passages at a split ratio of 1:2 using the manufacturers’ instructions. Total RNA was isolated using RNAeasy Plus Mini kit (Qiagen). For the reverse transcriptase reaction 2 μg RNA template was used to generate cDNA with Superscript II (Thermo Fisher Scientific) and random hexamers in a 20 μl reaction. One μl of the reverse transcriptase reaction was used as a template in a PCR reaction cycling between 94 °C (1′, initial denaturation) 94 °C (30”) −55 °C (1′) −72 °C (1′). Primers used in this study are shown in Table 2. Three independent RNA isolations and three different RT-PCR reactions were run as individual samples and analysed by agarose gel electrophoresis. Bands were visualized with SYBR Safe DNA gel stain (Thermofisher) and captured using autoexposure settings of a Biorad Chemidoc MP Imaging system.

**Table 2:** Primers used for RT-PCR

Target	Fragment size (bp)	Primer sequence (5′-> 3′)
CK-8	223	GAGGCATCACCGCAGTTAC TTGCTTCGAGCCGTCTTCT
KL-6	165	GTGCCGCCGAAAGAACTAC CTGCTGCCACCATTACCTG
E Cadherin	438	GGATTGCAAATTCCTGCCAT GAGTTCAGGGAGCTCAGACT
ENaC	300	CTCTGCTGGTTACTCACGAT AGTATCGGCTTCGGAACCTT
AQP3	296	TGACCAGTTCATAGGCACAGC ACACGAAGACACCCGCAAT
AQP5	233	GCCACCTTGTCGGAATCTAC CCAGTCCTCGTCAGGCTCATA
SIT1	454	CCAACTCGCTACAGTTCGTG AGGGACGGCGAGATATTGAG
ACE2	410	GATAAGCCTAAAATCAGCTC CAATGCCAACCACTATCACT
Clathrin	724	GACAGTGCCATCATGAATCC TTTGTGCTTCTGGAGGAAAGA

### Protein preparation, analysis, and Western blotting

For surface biotinylation cells were grown in 60-mm dishes and washed three times in 5 ml modified PBS (supplemented with 1 mM CaCl_2_ and 0.6 mM MgCl_2_, pH 8.0). Cells were then covered with 2 ml 0.5 mg/ml EZ-link Sulfo-NHS-lc-Biotin (Thermo Fisher Scientific) in modified PBS (pH 8.0) and incubated for 30 min at room temperature on a rotary shaker at low speed. Biotinylation was terminated by washing three times in modified PBS supplemented with 100 mM glycine, pH 8.0. Cells were scraped, collected in a 1.5-ml reaction tube and lysed by addition of 1 ml 150 mM NaCl, 1% Triton X-100, 20 mM Tris-HCl pH 7.5. The homogenate was incubated on ice for 1.5 h to complete lysis. Subsequently, the lysate was centrifuged at 13,000*g* in a table-top centrifuge for 10 min and the supernatant was transferred to a new tube. After protein determination, equal amounts of cell lysate were added to 150 μl high-capacity streptavidin agarose beads (Thermo Fisher Scientific). Beads were incubated overnight at 4 °C on a rotary shaker before washing four times in lysis buffer. The streptavidin-agarose slurry was mixed with protein sample buffer and reducing reagent. To prepare the protein samples for SDS-PAGE, 50 to 100 μg total protein was mixed with 4 × LDS sample buffer (Invitrogen), 10 × reducing agent (Invitrogen) and made up to a final volume of 40 μl using deionised water. Homogenised samples were then incubated at 70 °C for 10 min before being loaded on the gel. Electrophoresis was performed using 4 to 12% Bis-Tris polyacrylamide NuPAGE gels (Invitrogen), in an XCell *SureLock* Mini-Cell (Invitrogen) under reducing conditions according to standard procedures. The SeeBlue Plus 2 pre-stained protein ladder (Invitrogen) was used to estimate the apparent molecular weight of proteins. Following SDS-PAGE, proteins were transferred onto nitrocellulose membranes (GE Healthcare) using the Mini Trans-Blot Electrophoretic Transfer Cell (Bio-Rad) according to standard protocols. Blots were blocked for 2 h at room temperature (or overnight at 4 °C) in 50 ml 10% (w/v) skim milk in PBS (pH 7.4) with 0.15% TWEEN 20 (PBS–T). After washing thrice in PBS-T for 10 min each, the blots were incubated with the primary antibody overnight in 5 ml skim milk (2%, w/v) in PBS–T at dilutions listed in Table 3. Excess primary antibody was removed by washing thrice in PBS–T. Blots were incubated with 5 ml of diluted secondary antibody for 4 h. After washing thrice in PBS–T and a final rinse in PBS, immunoreactive bands were visualised by enhanced chemiluminescence, using Supersignal West Femto HRP substrate (Thermo Fisher) and captured using autoexposure settings of a Biorad Chemidoc MP Imaging system. For reprobing, the same blots were incubated for 30 min at 70 °C in 50 ml stripping buffer (62.5 mM Tris-HCl (pH 6.8), 2% SDS and 100 mM 2-mercaptoethanol). Membranes were then washed thrice with PBS-T and blocked for 3 h using 10% (w/v) skim milk in PBS-T before reprobing with the next antibody as described above. Antibodies used in this study are listed in Table 3. Specificity of antibodies was confirmed by expression of the antigen in *X. laevis* oocytes against a non-expressing control. Bands were quantified using the Gel analyses module of ImageJ.

**Table 3:** Antibodies used in this study

Target	Raised in species	Provider	Dilution
ACE2 (human)	Rabbit	Abcam ab15348 (1 mg/ml)	1:3000
SIT1 (mouse)	Rabbit	Custom Pineda Antibody service	1:500
Na/K-ATPase	Rabbit	Abcam ab76020	1:7500
IgG (rabbit)	Goat	Cell Signaling #7074	1:3000–1:10,000

## Data availability

The structures of the ACE2-SIT1 complex bound with tiagabine (PDB: 8WM3, whole map: EMD-33652; focused map: EMD-37868) have been deposited to the Protein Data Bank (http://www.rcsb.org) and the Electron Microscopy Data Bank (https://www.ebi.ac.uk/pdbe/emdb/), respectively.

## Supporting information

This article contains supporting information.

## Conflict of interest

The authors declare that they have no conflict of interest with the contents of this article.
