# Supplementary material for: Cryo-EM structure of ACE2-SIT1 in complex with tiagabine
Source: J Biol Chem. 2024 Aug 17;300(9):107687. doi: 10.1016/j.jbc.2024.107687 (PMC11414674; doi:10.1016/j.jbc.2024.107687)
Supplement: Supplemental Figures S1–S3 and Table S1 [file mmc1.pdf]

Supporting information

- Fig. S1
- Fig. S2
- Fig. S3
- Table S1

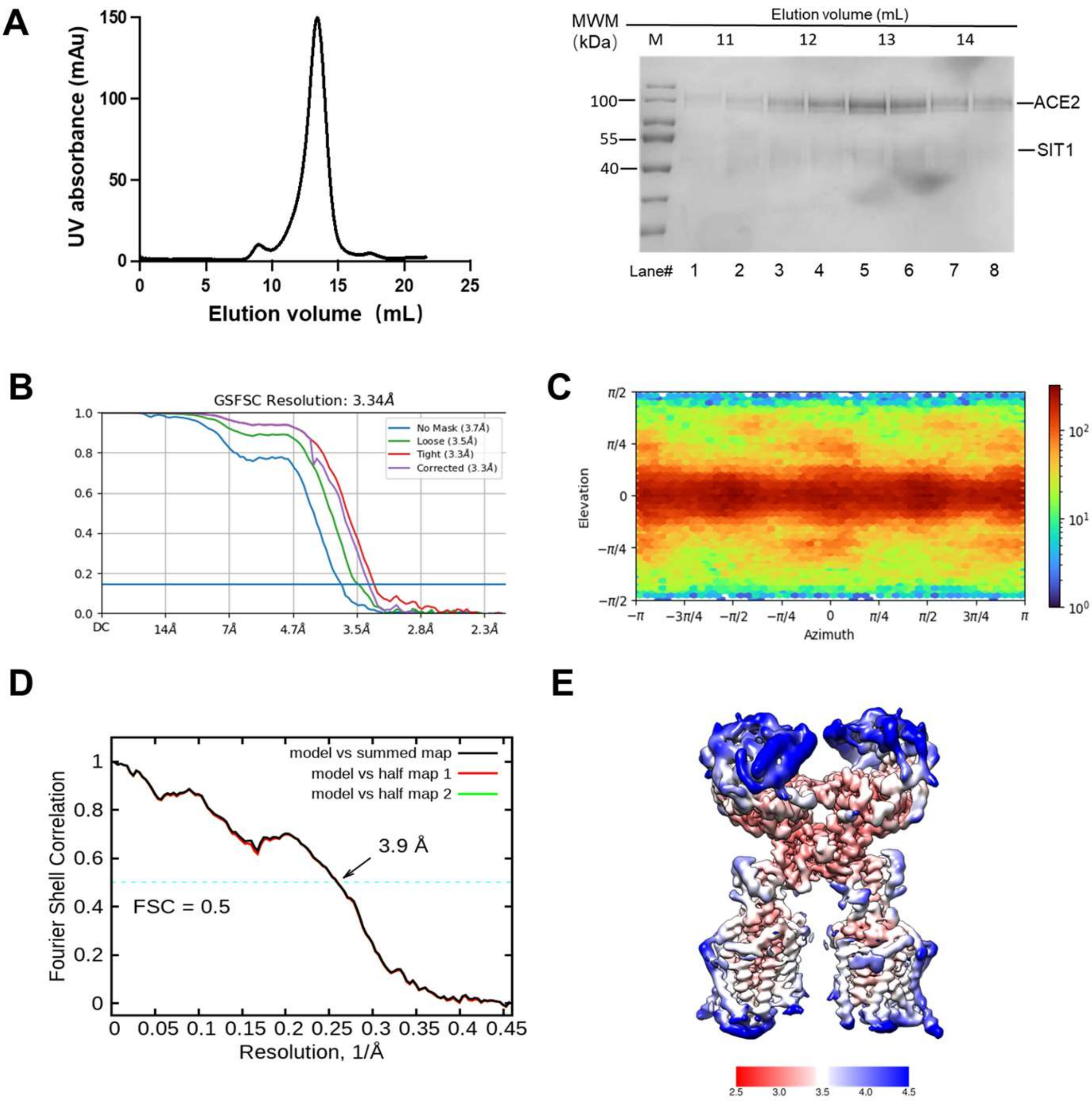

Figure S1. Cryo-EM reconstruction of ACE2-SIT1 bound with TGB.

(A) Representative size exclusion chromatography (SEC) purification of the ACE2-SIT1 complex. The protein complex was purified in the presence of GDN. Inset: on the left, the SEC purification diagram of the full-length human ACE2-SIT1 complex; on the right, SDS-PAGE visualized by Coomassie blue staining. MWM, molecular mass marker. **(B)** Gold-standard Fourier shell correlation (FSC) curves of the ACE2-SIT1 complex with TGI. The resolution was estimated with non-uniform refinement by CryoSPARC v3.1. **(C)** Euler angle distribution in the final 3D reconstruction of overall map. **(D)** FSC curve of the refined model versus the overall structure of class 2 that it is refined against (black); of the model refined against the first half map versus the same map (red); and of the model refined against the first half map versus the second half map (green). The small difference between the red and green curves indicates that the refinement of the atomic coordinates did not suffer from overfitting. **(E)** Cryo-EM density maps coloured by local resolution for ACE2-SIT1 bound with TGB**.**

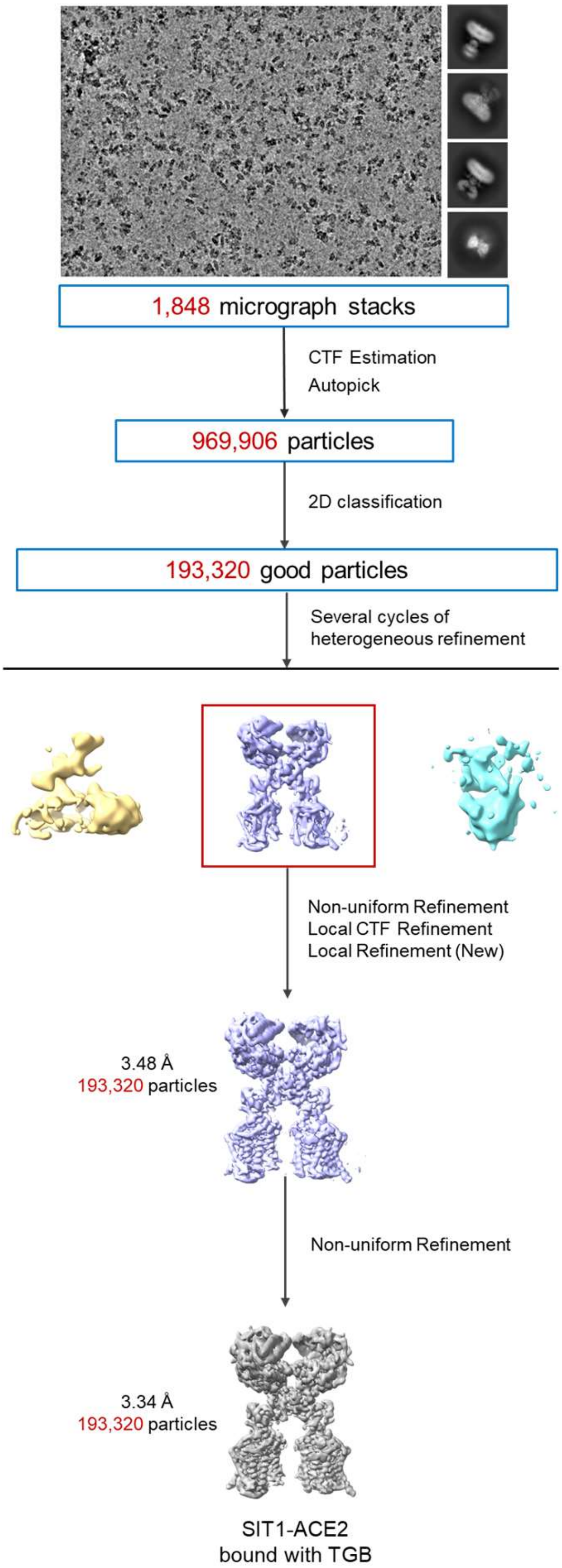

Figure S2. Flowchart for Cryo-EM data processing.

Please refer to the 'Data Processing' in Methods section for details.

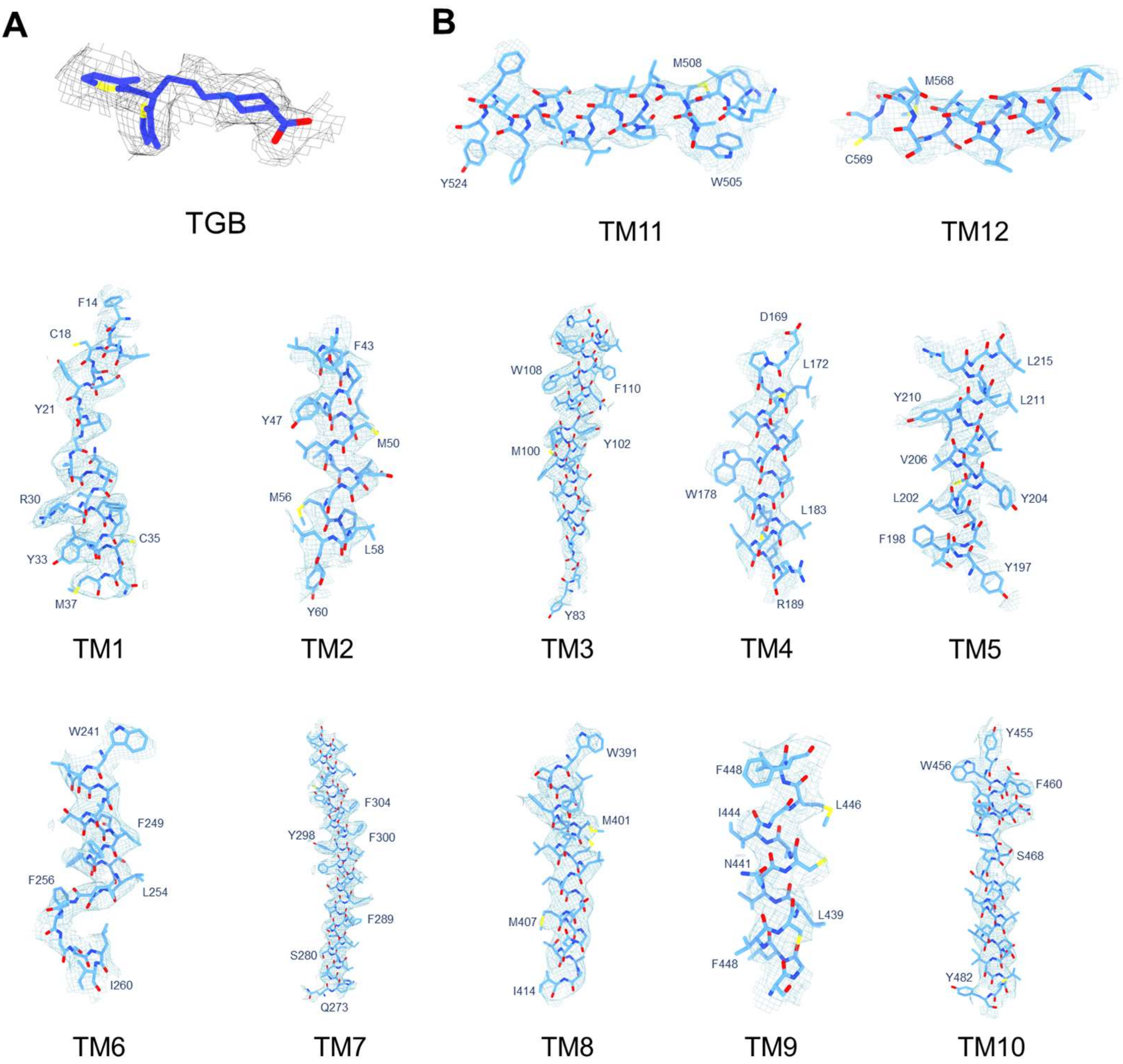

Figure S3. Cryo-EM density maps of the ACE2-SIT1 bound with inhibitors.

(a-b) Cryo-EM density maps for the inhibitors bound with SIT1 shown at counter level of 0.12. (**c-d)** Cryo-EM density maps for the transmembrane helix of SIT1 shown at counter level of 0.247.

Table S1 Cryo-EM data collection, refinement and validation statistics

Data collection	
EM equipment	Titan Krios (Thermo Fisher Scientific)
Voltage (kV)	300
Detector	Gatan K3 Summit
Energy filter	Gatan GIF Quantum, 20 eV slit
Pixel size (Å)	1.095
Electron dose (e-/Å2)	50
Defocus range (μm)	-1.4 ~ -1.8
Sample	ACE2-STI1 complex with TGB
Number of collected micrographs	1,814
3D Reconstruction	
Software	CryoSPARC
Number of used particles (Overall)	969,906
Resolution (Å)	3.34
FSC threshold for resolution determination	0.143
Symmetry	C1
Map sharpening B-factor ($Å^2$)	-90
Refinement	
Software	Phenix
Model composition	
Protein residues	2,642
Side chains assigned	2,642
CC-volume	0.85
CC-mask	0.84
B factors ($Å^2$)	151.5
R.m.s deviations	
Bonds length (Å)	0.005
Bonds Angle (˚)	0.731
MolProbitiy score	1.74
Ramachandran plot statistics (%)	
Preferred	95.86
Allowed	4.06
Outlier	0.04
